# Special Issue: Situated Psychology

**DOI:** 10.1007/s12124-025-09912-9

**Published:** 2025-05-19

**Authors:** Bo Allesøe Christensen, Noomi Matthiesen, Rasmus Birk, Sarah Kirkegaard Jensen, Svend Brinkmann, Thomas Szulevicz, Paula Cavada-Hrepich

**Affiliations:** Department of Communication and Psychology, Aalborg University, Aalborg, Denmark

## Introduction

Danish and Nordic psychology has historically been inspired by traditions emphasizing the historical, material, political, and cultural contexts of mental life, such as ecological psychology, activity theory, critical psychology, and community psychology. One significant idea shared by these disciplines is that psychological phenomena are situated, i.e. that the situation or context wherein these phenomena occur contributes to the constitution and understanding of these phenomena. This contrasts with another dominant idea in the history of psychology, namely the view that humans develop their psychological understanding initially from a distance to, or in separation from the surroundings in which they are embedded. Here the situation or context is often ignored, or at best considered as an add on to be considered ex post facto when analysing the process of understanding. Classic cognitive psychology, for example, analyses how sensory processes are processed by different cognitive processes before leading to some sort of action. The focus is more on analysing how this process takes place within the mind, than the context wherein this occurs. Situated psychology, in contrast, describes humans as intentional and meaning-making beings situated in historical, material, and social contexts. Here psychological processes are taken out of the laboratory and understood as not only occurring in the mind, but as part of ordinary contexts. These contexts are necessary elements of psychological phenomena and need to be considered and paid attention to as part of an analysis.

The psychology section at Aalborg University has been discussing the notion of Situated Psychology for years. A discussion initially started by Thomas Szulevicz and Svend Brinkmann. In 2022, they invited colleagues–from other Danish universities as well–to an ongoing discussion about the characteristics of such a psychology, both theoretically and methodologically. A first result of this work was published in the Danish journal *Nordiske Udkast (Nordic Outlines)* and the work published here builds on and extends that. This special issue investigates the central characteristics of a situated psychology through theoretical and empirical articles elaborating on central concepts and premises within situated psychology. The aim or hope of these investigations was never to create a finished or complete theoretical framework. Instead the aim was opening up a discussion by incorporating several perspectives on the anchoring of psychological phenomena in materiality, semiotics, and practice. The overarching point emerging across the contributing articles is that psychological phenomena like actions, thoughts, feelings, learning, development, and suffering have a practical character which cannot be separated from the social, historical, and material contexts within which these phenomena occur.

However, by addressing the situated nature of psychological phenomena, the authors aim to convey more than just the obvious, that psychological phenomena are social and relational. The concept of ‘situated’ underscores the need to combine perspectives that simultaneously focus on the material, semiotic, and active properties of human life’s anchoring in the world. Central to all contributions is therefore the dynamics and dialectic between participating social actors and the historical and concrete contexts in which they participate. These contexts are normatively structured with different expectations and ideals regarding how acts are possible and should be done. The situated social and material contexts within which humans engage in relation to someone and something are thus both *produced* by and simultaneously *producing* human actions, intentions, thoughts, feelings, experiences, and sufferings.

### The Articles

In the first article, “Situated Psychology: A Short Theoretical Sketch,” Rasmus Birk, Sarah Kirkegaard Jensen, Noomi Matthiesen, and Bo Allesøe discuss the historical roots of situated psychology. The article departs from William James’ pragmatism, which described psychology as a science of mental life and its conditions. Since its inception, psychology has been concerned with describing psychological phenomena as situated in specific conditions. The article also includes other perspectives that emphasize the situated aspects of the psychological. First, from anthropology, in the form of Jean Lave’s situated learning theory, and then from philosophy, where newer ideas about psychological externalism and 4E cognition are included. The article emphasizes that psychological phenomena should be understood based on human participation in concrete everyday practices and that the psychological should be understood as de-individualized and distributed among people. Finally, they argue that psychological phenomena are also historically created. The article thus describes a situated psychology, where social relations, normative structures, materiality, and historical and political processes are all central elements of human behavior, subjectivity, and being in the world.

Svend Brinkmann writes about a situated psychological understanding of psychopathology in the article “Situated Psychopathology.” The article is based on the recent history of psychiatry, which has seen a significant expansion of diagnoses. Since the etiological model of mental illness was replaced by the symptom model around 1980, new diagnoses have continuously been added to the current manuals, most recently with DSM-5 and ICD-11. The author elaborates on some of the criticism that has recently been directed at the expanding diagnostic psychiatry from two opposing perspectives: one represented by a neuroscientific approach known as Research Domain Criteria (RDoC), which argues that psychiatry should move beyond symptoms and find the causes of mental illness in the brain, and another represented by a contextual approach known as Power Threat Meaning Framework (PTMF), which argues that mental illness should be understood in light of what people experience in their lives. Brinkmann then argues that we need a situated perspective on mental illness that integrates neuroscientific knowledge about the brain and other aspects of the person with knowledge about the environment, as claimed by the PTMF approach. Such a perspective can be found in Dorte Gannik’s work on situated disease theory and, in another way, in Jerome Wakefield’s theory of harmful dysfunction, where mental illness in both cases is necessarily a property of a relationship between a person and an environment. The article’s aim is thus integrative, seeking to connect different newer theories that have conceptualized psychopathology in situated terms.

In the article “Situated Psychology as Triangulated: Understanding the Subjective, Intersubjective, and Objective as interrelated,” Bo Allesøe attempts to use the picture of triangulation as a framework for understanding the psychological character of situations as a relation between the subjective, intersubjective, and objective. Triangulation here is understood as a dynamic relationship that occurs internally to situations, where a subject, a person with a history, interacts/communicates with another person with a history and simultaneously relates to something objective or factual. Through analyses of, among others, Tomasello’s developmental psychology and the American philosopher Donald Davidson’s understanding of triangulation, a notion of the situated nature of psychological processes is developed. This is a non-reductive notion meaning that we fail to understand the complexity of psychological processes if the subjective, intersubjective, and objective are reduced to one another. A situated psychology should instead attempt to understand psychological phenomena as shaped by the interrelation between both personal, material, and social conditions.

Noomi Matthiesen and Paula Cavada-Hrepich focus on trust as a specific psychological phenomenon in the article “Windows of (dis)Trust: A Situated Psychological Perspective on Understanding the Phenomenon of Trust.” They analyze the significance of windows in daycare for trust between parents and educational professionals. The article is based on Knud Ejler Løgstrup’s radically relational and situated understanding of trust as a spontaneous and sovereign expression of life. They argue that in Løgstrup’s theory, there is an unresolved connection between the sovereign expression of life, which humans have not created themselves, and the normative social order with specific expectations and demands for participation. The article incorporates concepts from social practice theory to unfold this connection. They conclude the article by arguing that a situated psychology should address the aspects of social relationships in practice that demand particular ways of participating and simultaneously develop concepts to understand the part of life that are given vis-a-vis our humanity and thus beyond our control (as individuals and collectively).

In their article “Situating Educational Psychology Practice. Exploring the call for a ‘practice turn’ in contemporary Danish Educational Psychology Practice” Sarah Kirkegaard Jensen and Thomas Szulevicz explore a current debate concerning the professional identity and function of educational psychology (EP) in Denmark, with particular attention to the growing demand for a shift towards a more practice-oriented approach among educational psychologists. Through a situated psychological lens, the authors explore the requested practice turn of EP practice: what kind of practice is presented in the current educational and political discussion and what is the role of educational psychology and educational psychology practice in this raised critique of a ‘practice-distant’ EP practice? Through a situated psychological lens, the authors argue that understanding the field of EP practice, including the role of educational psychologists in contemporary educational practices, requires acknowledging its historical and contextual situatedness inside the practice of institutionalized education. In addition, the authors argue for a fundamental theoretical and normative discussion about the type of practices educational psychologists should support.

Tilde Lykke Mardahl-Hansen & Charlotte Højholt present in the article “Situated Teacher Professionalism: The Concept of Situated Inequality Highlights New Understandings of Teacher Professionalism and Conditions” a situated psychological understanding of teacher professionalism and teaching practice that addresses how teachers work with the complexities and dynamic interactions of everyday life as part of creating participation opportunities for children and young people in specific teaching situations. The authors’ starting point is a concept of ‘situated inequality,’ which invites understanding and analyzing inequality as simultaneously linked to the school as a particular societal practice, social interactions and collaboration processes, and the personal experiences, engagements, and lives of children and young people across places. It points to a connection between children’s and young people’s different opportunities to participate and succeed in school and professionals’ conditions for working with their participation opportunities in school. Drawing on practice theory, critical psychological concepts such as conduct of everyday life, and Donald Schon’s theory of professionalism in practice, the authors present an understanding of teacher professionalism as situated in the social everyday life of the school, where teachers and students collaborate on differences and common tasks. The aim is to strengthen understandings and conditions for teachers’ professionalism in working with children’s and young people’s conditions for participation in the school’s learning communities.

### Findings

What emerges is a picture of a situated psychology striving to find theoretical orientations and concepts complex enough to capture and describe psychological phenomena as they unfold in everyday life, which is deeply entangled, composite, and dynamic. We emphasize that this is a project still in the process of becoming and developing. The intentions with this special issue have been to unfold a range of understandings of situated psychology, and while we believe there is potential to further develop a situated psychology, there are also several unresolved discussions that need to be addressed. What are the possibilities for situated psychology moving forward, both theoretically and methodologically? How does situated psychology relate to some of the other traditions within qualitative psychological research? What new types of questions and perspectives does situated psychology contribute with?

With this special issue, we hope to invite others into the work of continuing to develop a situated psychology.

## Data Availability

No datasets were generated or analysed during the current study.

